# Improving the enzymatic hydrolysis of thermo-mechanical fiber from *Eucalyptus urophylla* by a combination of hydrothermal pretreatment and alkali fractionation

**DOI:** 10.1186/s13068-014-0116-8

**Published:** 2014-08-20

**Authors:** Shaoni Sun, Xuefei Cao, Shaolong Sun, Feng Xu, Xianliang Song, Run-Cang Sun, Gwynn Lloyd Jones

**Affiliations:** Beijing Key Laboratory of Lignocellulosic Chemistry, Beijing Forestry University, Beijing, 100083 China; State Key Laboratory of Pulp and Paper Engineering, South China University of Technology, Guangzhou, 510640 China; School of Natural Science, University of Wales, Gwynedd, Bangor, LL57 2UW Wales UK

**Keywords:** Eucalyptus fiber, Hydrothermal pretreatment, Alkali fractionation, Enzymatic hydrolysis

## Abstract

**Background:**

The recalcitrance of lignocellulosic biomass is a major limitation for its conversion into biofuels by enzymatic hydrolysis. The use of a pretreatment technology is an essential step to diminish biomass recalcitrance for bioethanol production. In this study, a two-step pretreatment using hydrothermal pretreatment at various temperatures and alkali fractionation was performed on eucalyptus fiber. The detailed chemical composition, physicochemical characteristics, and morphology of the pretreated fibers in each of the fractions were evaluated to advance the performance of eucalyptus fiber in enzymatic digestibility.

**Results:**

The hydrothermal pretreatment (100 to 220°C) significantly degraded hemicelluloses, resulting in an increased crystallinity of the pretreated fibers. However, as the pretreatment temperature reached 240°C, partial cellulose was degraded, resulting in a reduced crystallinity of cellulose. As compared to the hydrothermal pretreatment alone, a combination of hydrothermal and alkali treatments significantly removed hemicelluloses and lignin, resulting in an improved enzymatic hydrolysis of the cellulose-rich fractions. As compared with the raw fiber, the enzymatic hydrolysis rate increased 1.1 to 8.5 times as the hydrothermal pretreatment temperature increased from 100 to 240°C. Interestingly, after a combination of hydrothermal pretreatment and alkali fractionation, the enzymatic hydrolysis rate increased 3.7 to 9.2 times. Taking into consideration the consumption of energy and the production of xylo-oligosaccharides and lignin, an optimum pretreatment condition was found to be hydrothermal pretreatment at 180°C for 30 min and alkali fractionation with 2% NaOH at 90°C for 2.5 h, in which 66.3% cellulose was converted into glucose by enzymatic hydrolysis.

**Conclusions:**

The combination of hydrothermal pretreatment and alkali fractionation was a promising method to remove hemicelluloses and lignin as well as overcome the biomass recalcitrance for enzymatic hydrolysis from eucalyptus fiber. In addition, the various techniques applied in this work constituted an efficient approach to understand the underlying chemical and morphological changes of the cellulose-rich fractions.

**Electronic supplementary material:**

The online version of this article (doi:10.1186/s13068-014-0116-8) contains supplementary material, which is available to authorized users.

## Background

Lignocellulosic biomass, the most abundantly renewable resource in the world, is a promising alternative to fossil resources for the production of energy, materials, and chemicals. However, lignocellulosic biomass is recalcitrant to biodegradation due to the rigid and compact structure of the plant cell walls (known as “biomass recalcitrance”) [1]. Biomass recalcitrance mainly results from the hard-packed spatial network that serves as a protective bulwark [1]. The presence of substances such as hemicelluloses, lignin, and pectin and their spatial inter-links construct physical barriers to protect cellulose from degradation. It has been found that the factors affecting cellulose conversion include lignin, hemicelluloses, the crystallinity and polymerization degree of cellulose, specific surface area, and pore volume, among others [2,3]. Thus, an effective pretreatment technology is crucial to reduce the recalcitrance and make cellulose more available to enzymatic attack.

Different pretreatment methods exist, each with unique action mechanisms, such as removing hemicelluloses and/or lignin, reducing the cellulose crystallinity, and increasing the biomass surface area. An efficient pretreatment technology should also maximize cellulose recovery from the pretreatment process. Among the various pretreatment methods, hydrothermal treatment combined with alkali treatment is a potential process for effectively fractionating lignocellulosic biomass. According to a biorefinery concept, the process can disrupt the hemicellulose-lignin complex and separate the biomass into main components for further utilization [4,5]. Hydrothermal pretreatment has been considered as an environmentally friendly processing technology, since the medium only contains feedstock and water, thus avoiding many problems, such as corrosion and acid recycling [6,7]. In particular, hydrothermal pretreatment can efficiently convert hemicelluloses into soluble compounds that are mainly composed of mono- and oligosaccharides, which could be further hydrolyzed into value-added chemicals. The solid phase is rich in cellulose and lignin, from which lignin is not removed effectively. As we know, lignin is deemed a significant element affecting enzymatic hydrolysis efficiency due to its high adsorption ability for enzymes [8]. Alkali fractionation has been implemented to effectively solubilize lignin and destroy the rigid structure of lignocellulosic biomass, increasing the availability of cellulose to enzymes. Among various alkalis, sodium hydroxide (NaOH) exhibits the most significant effect on degrading lignin and improving subsequent fermentation yields [9,10]. The use of a NaOH aqueous solution can swell the plant cell walls and disrupt the lignin structure, reduce the degree of polymerization and crystallinity of cellulose, and also increase the surface area of cellulose [11].

**Figure 1:**
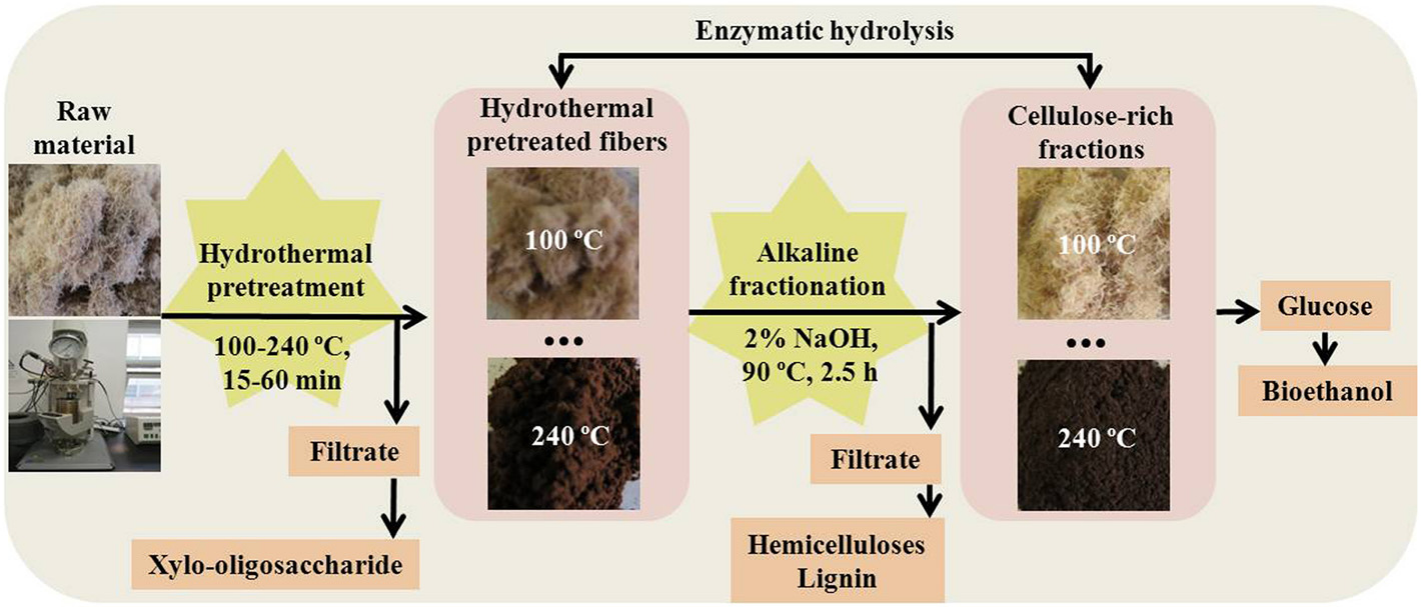
**Schematic representation of the processing of eucalyptus fiber by hydrothermal pretreatment and alkali fractionation.**

In the present study, a two-step consecutive pretreatment was applied to thermo-mechanical fiber from *Eucalyptus urophylla* to reduce the cellulose recalcitrance and thus improve the enzymatic hydrolysis efficiency. This process included hydrothermal pretreatment under different conditions (temperature and time) predominantly to remove hemicelluloses, followed by alkali (NaOH) fractionation to remove lignin. The hydrothermal pretreated fibers and cellulose-rich fractions were then subjected to enzymatic hydrolysis to evaluate the effect of the hydrothermal and alkali treatments. The chemical composition, physicochemical characteristics, morphology, and enzymatic digestibility of the pretreated samples were detected in order to understand the chemical and structural changes of the cellulose fiber. Figure 1 shows the schematic representation of the hydrothermal pretreatment and alkali fractionation of eucalyptus fiber used in this work.

## Results and discussion

### Mass loss and chemical composition of the pretreated fibers and inhibitors in hydrothermal liquor

**Figure 2:**
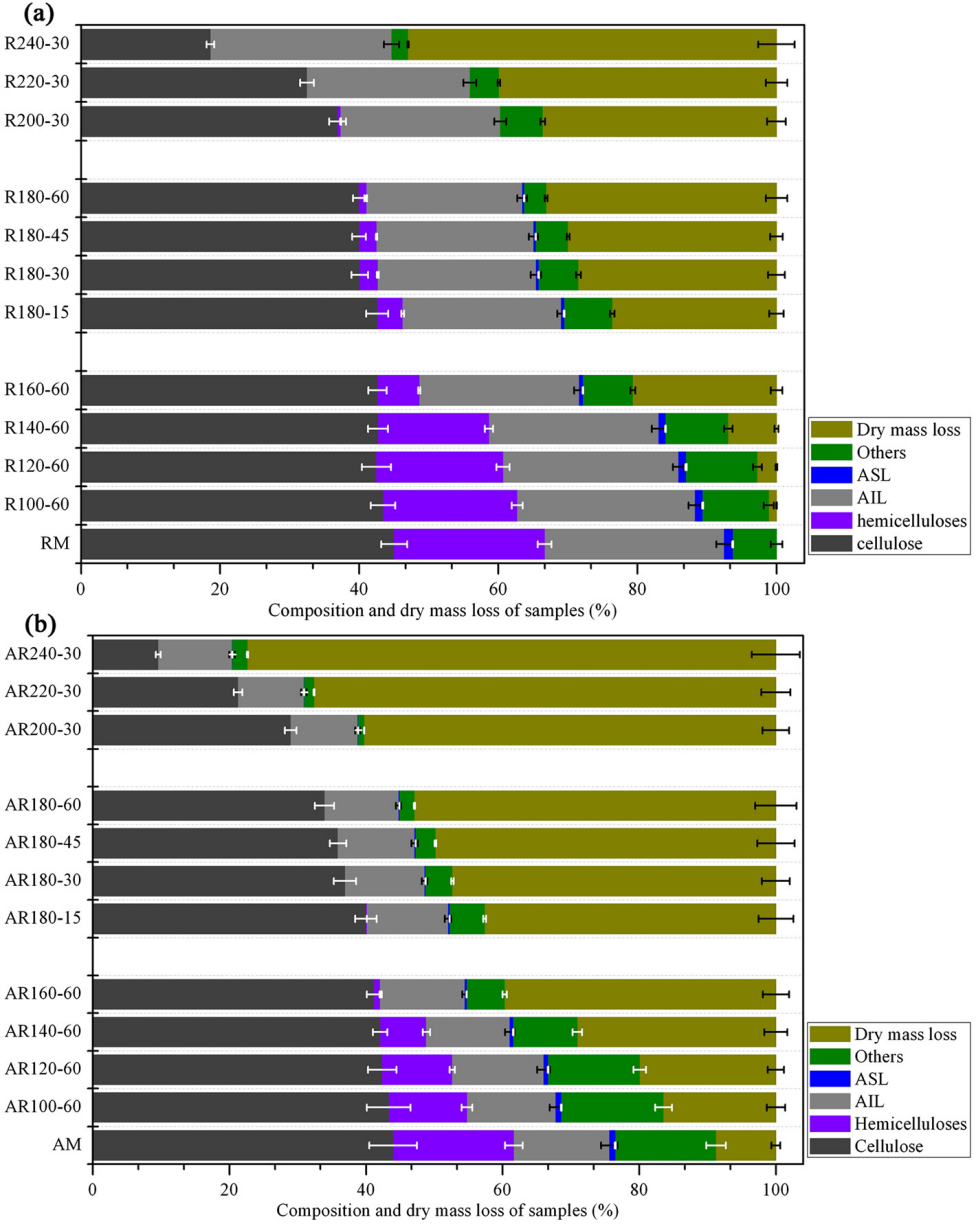
**Composition (% dry mass) and dry mass loss of hydrothermal pretreated fibers (a) and synergistic pretreated samples (b).** RM: raw material; R: residues obtained after the direct hydrothermal pretreatment of the raw material; AM: residue obtained after the direct alkali treatment of the raw material without hydrothermal pretreatment; AR: residues obtained after the combination of hydrothermal pretreatment and alkali treatment of the raw material. For chemical compositions of the raw fiber, hydrothermal pretreated fibers, and the cellulose-rich fractions obtained after synergistic treatment, see Additional file 1: Table S1.

Hydrothermal pretreatment was utilized to remove hemicelluloses and break up the compact structure of the biomass. As shown in Figure 2, the dry mass loss increased gradually with temperature. After pretreatment at 100°C for 60 min (R_100-60_), only 1.1% biomass was consumed by hot water, but more than 50% biomass was lost after pretreatment at 240°C for 30 min (R_240-30_). As expected, hemicelluloses remaining in the pretreated samples decreased with an increase of temperature and almost disappeared when the pretreatment temperature was higher than 200°C (Additional file 1: Table S1). Ibbett and Merali *et al.* [12,13] reported that hemicelluloses were dissolved as small molecular weight oligomers during hydrothermal pretreatment. At a high temperature, water autoionization generates hydronium ions (H_3_O^+^), leading to hydrolysis and deacetylation of hemicelluloses, in which H_3_O^+^ is further generated from the released acetic acid to improve hemicellulose degradation. Compared to the hemicelluloses, when the temperature was below 200°C, the cellulose and total lignin contents in the hydrothermal pretreated fibers were reduced slightly. As the temperature further increased, the percentage of cellulose removal rose markedly and reached a maximum value (58.7%) at 240°C. After hydrothermal pretreatment (100 to 180°C), the cellulose content in the residues was increased continuously from 43.91% to 59.69% (Additional file 1: Table S1). The organic acids formed during the hydrothermal pretreatment might catalyze the hydrolysis of glycosidic bonds in hemicelluloses to mono- and oligosaccharides and thereby relatively increase the cellulose content. Furthermore, the removal of hemicelluloses from the surface of cellulosic microfibers might also lead to an increment of cellulose pore volume. However, when the pretreatment temperature reached 200°C, the content of cellulose decreased, which was probably due to the degradation of small amounts of cellulose under the harsh conditions. The lignin content in the pretreated residues increased from 25.84 to 54.35% with increasing temperature (Additional file 1: Table S1), which was mainly due to the removal of hemicelluloses. It should be noted that a similar degree of removal of cellulose and acid insoluble lignin (AIL) was observed under mild conditions (temperature < 200°C). However, when the pretreatment temperature increased above 200°C, cellulose was more susceptible to the pretreatment temperature. The hydrothermal pretreatment at 100 to 180°C resulted in 0.9 to 13.2% lignin removal. The noticeable degradation of lignin was presumably a crucial prerequisite for the efficient removal of hemicelluloses, since lignin was covalently associated with hemicelluloses. In contrast, lignin removal decreased sharply to 0.9% at 240°C, which was due to the condensation of lignin and precipitation of the dissolved lignin fragments on the surface of cellulose [14]. Furthermore, the percentage of removal of AIL and acid soluble lignin (ASL) was significantly different. Generally, ASL is composed of low molecular weight lignin and secondarily formed hydrophilic materials, showing less carbon content than AIL [15,16]. Therefore, the difference in the percentage of removal between AIL and ASL might suggest that the lignin structure had been altered during the hydrothermal pretreatment, for example, by linkage cleavage and depolymerization [17,18].

As previously stated, the synergistic effect of hydrothermal pretreatment and alkali fractionation could improve the solubilization of hemicelluloses and lignin [19]. A greater dry mass loss of the hydrothermal pretreated sample than that of the raw material was observed after the alkaline treatment (Figure 2b), which is probably ascribed to the fact that more reactive sites and accessible area were released during the hydrothermal pretreatment. During the alkaline treatment, cleavages of hydrolyzable linkages, such as alpha- and beta-aryl ethers in lignin and glycosidic bonds in carbohydrates, constitute the primary reactions that lead to the dissolution of some lignin and carbohydrates [20]. Specifically, the dry mass losses of hemicelluloses and cellulose were attributed to the “peeling” and hydrolytic reaction, while the enhanced delignification was probably due to the depolymerization and modification of lignin macromolecules [18]. This effect eliminated the nonproductive adsorption sites of cellulose and then increased the accessibility. The combination of hydrothermal pretreatment and alkali fractionation resulted in the AIL and ASL decreasing from 25.84 to 54.35% and 0.42 to 1.12% to 15.06 to 47.79% and 0.15 to 0.96%, respectively (Additional file 1: Table S1). Based on the above results, a combination of hydrothermal and alkali treatments could remove more hemicelluloses and lignin than the hydrothermal pretreatment alone, thus resulting in a high glucan content in the cellulose-rich fractions, which would facilitate the subsequent enzymatic saccharification.

During the hydrothermal pretreatment process, some inhibitors were generated, especially at higher pretreatment temperatures. The concentrations of the main inhibitors, including acetic acid, furfural, and 5-hydroxymethylfurfural (HMF), are listed in Additional file 2: Table S2. Acetic acid was formed as a consequence of hydrolysis of the acetyl groups from the hemicellulosic polymers, while furfural and HMF were generated from the dehydration of pentose and hexose, respectively. The concentration of furfural was always higher than that of HMF, which was attributed to the easier dehydration of pentose. As expected, the concentrations of the three inhibitors increased with increasing temperature and time, and their maximal concentrations were observed at 240°C for 30 min, corresponding to the harshest experimental conditions in this work. It should be noted that these inhibitors, under the concentration levels given, did not induce any significant inhibition of the activity of the various fermentative microorganisms, since the solid residue was made almost free of inhibitors by thoroughly washing it with hot water and by following the hydrothermal pretreatment with the alkali treatment.

### Physicochemical characteristics of the hydrothermal pretreated fibers and the cellulose-rich fractions obtained after the synergistic treatment

Fourier transform infrared spectroscopy (FTIR) analysis was utilized to investigate the effects of hydrothermal pretreatment and alkaline fractionation on the chemical structure of biomass (Additional file 3: Figure S1). Characteristic assignments of hemicelluloses at 1738 (C = O conjugates in xylan) and 1244 (C-O) cm^-1^ were observed in the raw material. The decline of the intensities of the two bands with temperature (100 to 220°C) was indicative of the dissolution/removal and/or deacetylation of hemicelluloses [21]. When the hydrothermal pretreatment condition became severe (240°C), the two peaks almost disappeared, confirming the significant degradation of hemicelluloses. Also, the disappearance of the band at 1738 cm^-1^ in all of the alkali-treated samples revealed that alkaline treatment cleaved the ester bands of hemicelluloses, such as acetyl and uronic ester groups [22]. The intensity of the peak at 1506 cm^-1^ (skeletal vibrations of the lignin aromatic rings) increased with the increment of the pretreating temperature and time, which was attributed to an increase of the lignin concentration in the hydrothermal pretreated fibers. Additionally, a decreasing intensity of this peak in the cellulose-rich fractions was probably due to the significant removal of lignin during the alkaline fractionation process. A slight decline in the intensity of the peak at 1373 cm^-1^ (C-H deformation in cellulose and hemicelluloses) was observed as a function of temperature and of time. Moreover, a decreasing intensity at 1244/1232 cm^-1^ originating from C-O bending vibration in hemicelluloses indicated the decreasing hemicellulose content, which was in accordance with the results obtained by chemical composition analysis. However, a band at around 1036 cm^-1^, attributed to guaiacyl units of lignin, was observed and gradually increased with temperature. Also, the intensity of the typical cellulose peak at 1161 cm^-1^ rose as the temperature increased, which resulted from the removal of hemicelluloses and lignin [23].

The crystallinity index (CrI) values of the untreated and pretreated fibers were calculated (Table 1), and their X-ray diffraction (XRD) patterns are shown in Additional file 4: Figure S2. The CrI of the untreated fiber, containing a large amount of amorphous components (mainly hemicelluloses and lignin), was 57.02%. The CrI successively increased with temperature (100 to 220°C), which could be attributed to the removal of hemicelluloses, as confirmed by the chemical composition and FTIR analysis. However, the CrI value of R_240-30_ decreased, which was mainly attributed to the partial degradation of crystal cellulose and extensive condensation and re-precipitation of lignin on the surface of cellulose compared to the other samples. Moreover, as compared to the untreated sample, the alkali-treated sample without hydrothermal pretreatment exhibited a high CrI (63.06%). When the hydrothermal pretreated residues (AR_100-60_ to AR_240-30_) were subjected to alkali treatment, their crystallinities increased slightly (63.06 to 72.94%) compared to those of R_100-60_ to R_240-30_ (57.52 to 71.75%). The increase in CrI was mainly attributed to the increase in cellulose concentration of the pretreated sample because of the removal of hemicelluloses and lignin (ranging from 25.79 to 54.35% to 16.98 to 47.79%) under the alkali conditions. Chen *et al.* [24] reported that NaOH treatment increased the CrI because certain amounts of amorphous materials (xylan and lignin) were dissolved by the NaOH treatment.

**Table 1:** **The crystallinity indexes of the raw fiber, hydrothermal pretreated fibers, and the cellulose-rich fractions obtained after the synergistic treatment**

	**XRD-CrI (%)**	**NMR-CrI (%)**		**XRD-CrI (%)**	**NMR-CrI (%)**
RM	57.02	43.71	AM	59.79	46.45
R_100-60_	57.52	45.26	AR_100-60_	63.06	46.47
R_120-60_	58.31	46.39	AR_120-60_	65.24	46.66
R_140-60_	60.83	46.79	AR_140-60_	66.30	47.22
R_160-60_	62.98	49.69	AR_160-60_	66.75	50.74
R_180-15_	67.04	50.59	AR_180-15_	67.90	52.73
R_180-30_	68.05	51.48	AR_180-30_	68.23	52.83
R_180-45_	68.15	51.71	AR_180-45_	69.23	52.97
R_180-60_	69.78	53.45	AR_180-60_	69.26	53.53
R_200-30_	70.88	54.41	AR_200-30_	69.91	54.37
R_220-30_	71.75	55.79	AR_220-30_	72.94	54.82
R_240-30_	68.74	53.86	AR_240-30_	71.53	50.75

**Figure 3:**
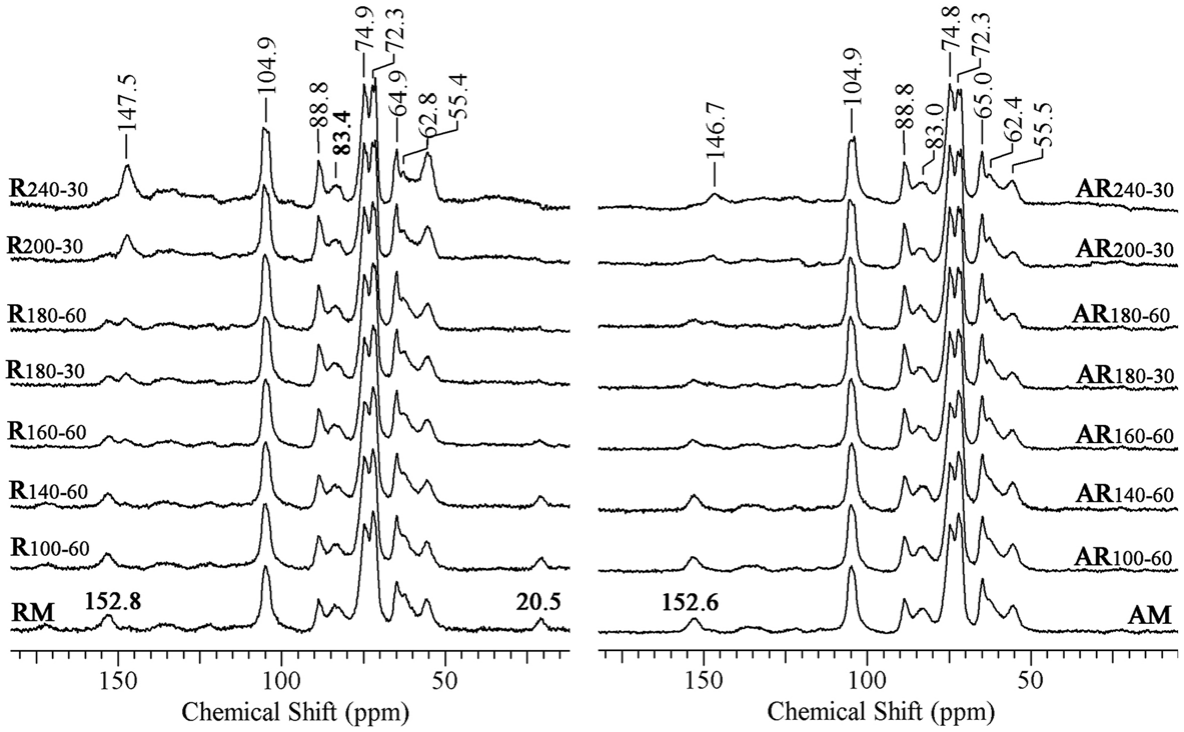
**CP/MAS** ^**13**^ **C NMR spectra of the raw fiber, hydrothermal pretreated fibers, and the cellulose-rich fractions.**

Further information on structural changes in the samples that occurred as a consequence of hydrothermal pretreatment and alkali fractionation was obtained by high resolution solid state cross-polarization/magic angle spinning ^13^C nuclear magnetic resonance (CP/MAS ^13^C NMR) (see Figure 3). The signals at 88.8 and 64.9 ppm originate from C4 and C6 carbons from the crystalline interior of cellulose microfibrils, whereas the peaks at 83.4 and 62.8 ppm are assigned to C4 and C6 carbons in less-ordered cellulose on the surface of microfibrils and hemicellulose [25]. The intensities of C4/C6 signals of the less-ordered cellulose declined slightly with temperature, suggesting that considerable removal of amorphous cellulose and hemicelluloses occurred during the pretreatment process. In this work, the ratio of the area of the 86 to 92 ppm to 80 to 92 ppm region was also calculated as crystallinity [26]. The CrI values of samples from the two measurement techniques were different. Park *et al.* [27] reported that the crystallinity from the XRD peak height method was higher than that obtained from the CP/MAS ^13^C NMR method, since NMR technology takes into account the cellulose chains present on the surface of cellulose crystals. However, a similar trend could be observed for these samples; that is, CrI successively increased with temperature from 100 to 220°C but sharply decreased at 240°C (Table 1).

Signals from lignin are concentrated in the region of 100 to 200 ppm and are relatively broad due to the chemical complex and disordered structure. Signals for lignin were observed at 152.8 (syringyl lignin), 147.5 (guaiacyl lignin), and 55.4 (methoxy groups) ppm, respectively. The decreasing intensity at 152.8 ppm and increasing intensity at 147.5 ppm implied that the relative content of syringyl units of lignin in the hydrothermal pretreated samples decreased, while that of the guaiacyl units increased. Moreover, after the alkali treatment, the intensity of the guaiacyl-units signal was reduced significantly, suggesting that alkali fractionation had an active role in the removal of guaiacyl lignin. Coinciding with the changes in chemical composition, the lignin signals of R_240-30_ were more pronounced and differentiated compared to the other samples, which was closely related to the recalcitrance of lignin to the autohydrolysis at a high temperature. Also, the spectrum of RM showed two weak peaks at 20.5 and 172.1 ppm for methyl and carboxylic carbons of acetyl groups attached to hemicelluloses [28,29], and the intensities of the two resonances decreased with the degradation of hemicelluloses at higher temperature, as confirmed by FTIR analysis. Moreover, the two signals disappeared in the alkali-treated samples, implying the removal of acetyl groups in hemicelluloses. Peng *et al.* [30] reported that the cleavage of *O*-acetyl groups occurred and all acetyl groups were split off under alkali conditions. The cleavage of acetyl groups might reduce the steric obstacles of hemicelluloses for enzymatic hydrolysis, possibly increasing the sugar conversion in enzymatic hydrolysis [31,32].

### Morphological analysis

**Figure 4:**
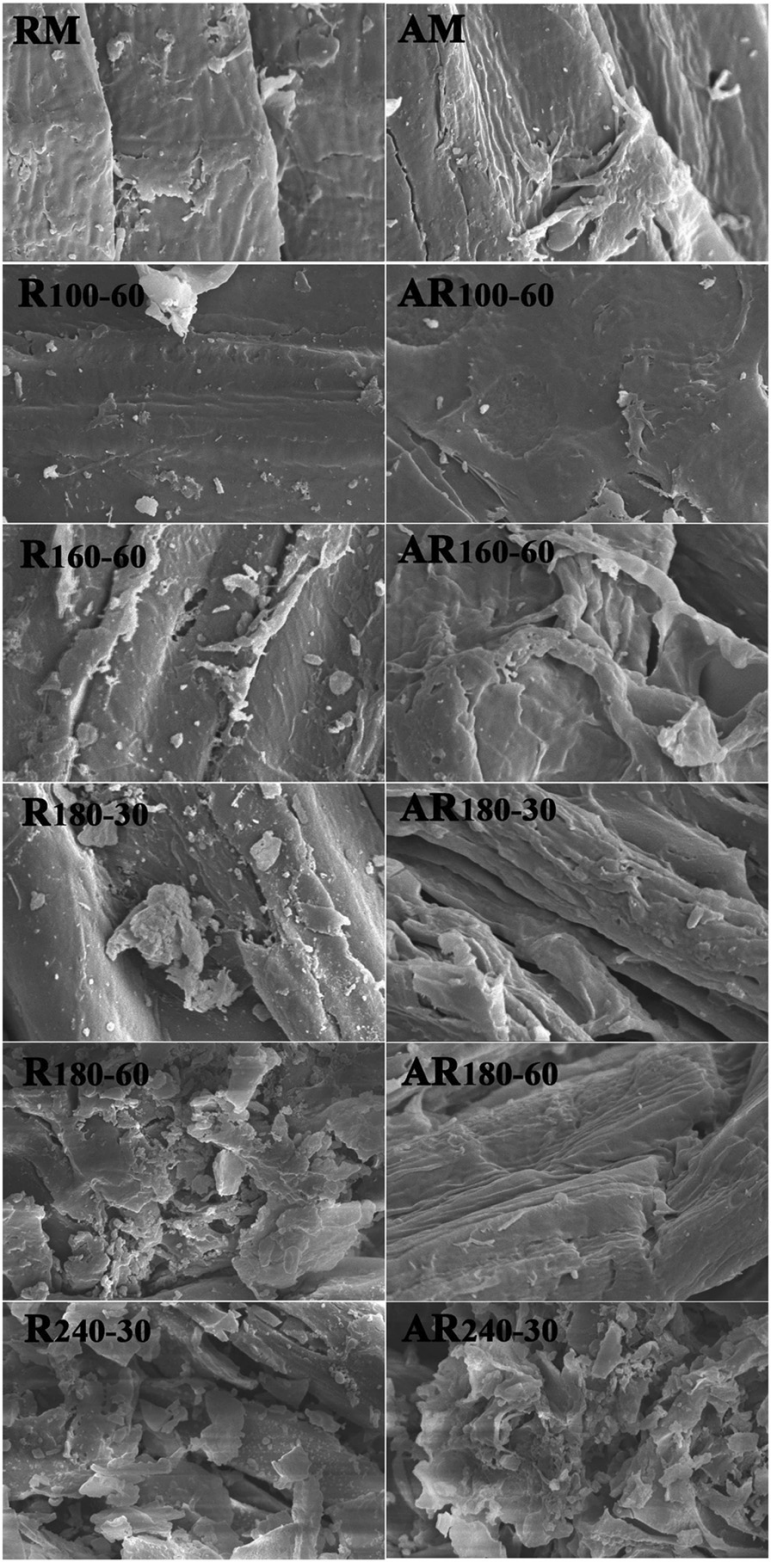
**SEM images of the raw fiber, hydrothermal pretreated fibers, and the cellulose-rich fractions at magnification × 3000.**

Scanning electron microscopy (SEM) images of the biomass before and after hydrothermal pretreatment and alkali treatment are shown in Figure 4. As can be seen, the RM displayed a compact and regular surface structure and fibers arranged in bundles, which impeded the accessibility of cellulase to cellulose. The hydrothermal pretreatment resulted in evident damage to the plant cell structure, which would benefit enzymatic digestibility of the pretreated fiber. The morphology of R_120-60_ was similar to that of RM because of the limited impact of pretreatment under the mild conditions. At higher temperatures (160°C for 60 min and 180°C for 30 min), the surfaces of the samples were partially destroyed, as shown by the presences of cracks and small debris. A possible reason was the partial removal of hemicelluloses and lignin during the hydrothermal pretreatment. Under harsher conditions (240°C), the SEM image shows well-broken fibers and an increased surface area as compared with the other hydrothermal pretreated samples.

Clearly, there was no obvious change in the surface structure between RM and AM. After a low temperature pretreatment (100°C), the alkali-treated sample AR_100-60_ exhibited slightly morphological changes on the surface of the fibers. When the pretreatment temperature increased to 160 and 180°C, some minor debris on the fiber surfaces was removed, and the surface structure of the alkali-treated samples tended to be smooth, resulting in the exposure of more fiber bundles; thus, the accessibility of fiber bundles to cellulase could be improved. After pretreatment at 240°C, the cellulose structure of AR_240-30_ was damaged and more small pieces were generated. Overall, the hydrothermal pretreatment shows an obvious effect on hemicellulose removal, while the following alkali fractionation can remove lignin effectively. The removal of both hemicelluloses and lignin resulted in the release of a larger specific surface area, which favored the following enzymatic hydrolysis.

### Enzymatic hydrolysis

**Figure 5:**
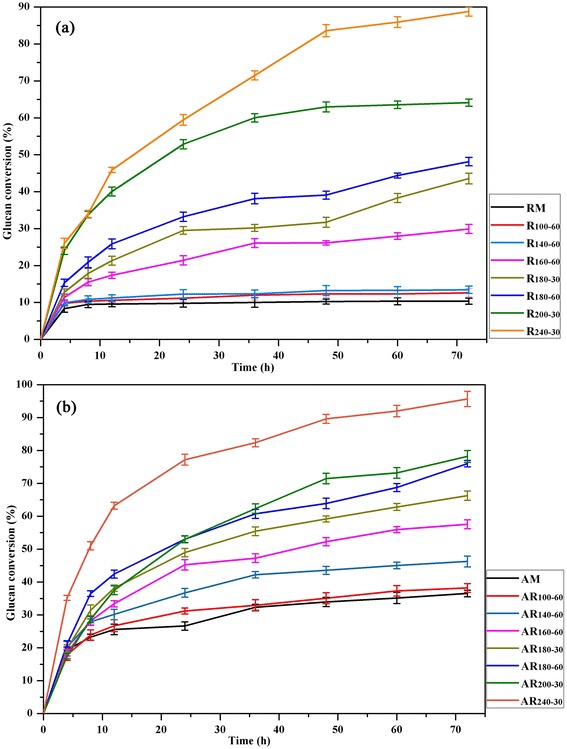
**The enzymatic hydrolysis of the fibers after hydrothermal pretreatment (a) and the synergistic treatment (b).** The error bars are standard deviations from the average values of duplicate determinations.

Figure 5 shows the sugar yields from the enzymatic saccharification of the native and pretreated fibers. As expected, the cellulose conversion increased with increasing pretreatment temperature. After 72 h of hydrolysis, only 10.36% of glucan was converted into glucose for RM. The conversion rates of the hydrothermal pretreated samples at low temperature (≤140°C) were 11.94 to 13.44%. The alkaline treatment led to a substantial enhancement in enzymatic hydrolysis (38.21 to 46.26%). However, the saccharification of the sample AM (treated with alkali alone) was 36.52%, which was higher than those of RM. At 160 to 180°C, higher glucose yields were obtained and the glucose release progressively increased with the pretreatment temperature and time. This enhanced enzymatic hydrolysis was likely attributed to the effective removal of hemicelluloses and the partial removal of lignin, as identified by the data in Figure 2. Lignin affected the enzymatic digestibility effectiveness by inhibiting enzyme function or by acting as an enzymatic trap, which led to an unproductive adsorption of enzymes [33]. Meanwhile, the SEM images showed that hemicellulose and lignin removal led to generations of cracks and small debris on the cellulose fibers, which enhanced the accessibility of substrate to enzyme. After the sequential NaOH treatment, the enzymatic hydrolysis of the cellulose-rich fractions was significantly improved, which demonstrated that the sequential alkali treatment was beneficial for increasing glucose conversion. Alkali fractionation enhanced the removal of hemicelluloses and lignin from the fibers, reducing the physical barrier and the invalid adsorption of cellulase on lignin. The effective adsorption of cellulase on cellulose, which is the first step in the hydrolysis reaction and a precondition for efficient cellulose conversion [17,34], is closely related to the accessible surface area of cellulose [35]. When the pretreatment temperature increased to 220°C or even 240°C, the enzymatic hydrolysis efficiency of the alkali-treated residue was significantly enhanced compared with the others. The maximal glucose yield of 95.6% with a low cellulose recovery (21.3%) was achieved by the combination of hydrothermal pretreatment at 240°C for 30 min and 2% NaOH treatment; this yield was increased by 8.2 times compared with that of the raw material (10.4%). The high glucose yield was likely due to the serious disintegration of cellulose (as shown in the SEM image), which provided a larger surface area and larger amounts of cellulose chain ends and thus improved enzyme accessibility.

### Process mass balance

**Figure 6:**
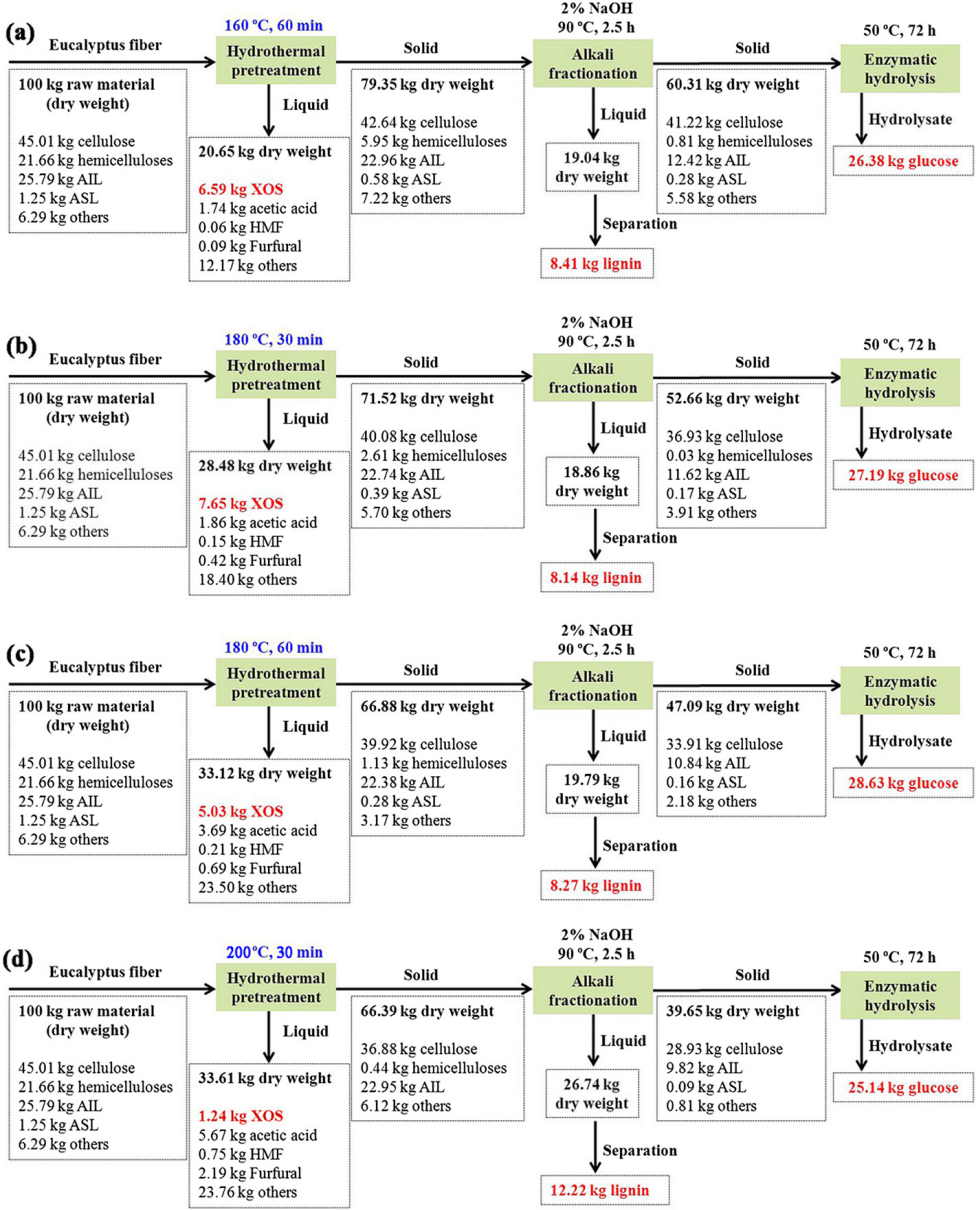
**Material balances during the synergistic treatment and enzymatic hydrolysis for the four pretreatment conditions. (a)** Hydrothermal pretreatment at 160°C for 60 min combined with the alkali treatment, **(b)** hydrothermal pretreatment at 180°C for 30 min combined with the alkali treatment, **(c)** hydrothermal pretreatment at 180°C for 60 min combined with the alkali treatment, and **(d)** hydrothermal pretreatment at 200°C for 30 min combined with the alkali treatment.

A process mass balance of the two-step pretreatment process and the subsequent enzymatic hydrolysis was developed for the four hydrothermal pretreatment conditions (Figure 6). Process yields were normalized to a common basis of 100 kg of dried eucalyptus fiber as the starting material. After the two-step pretreatment process, the yields of the residual eucalyptus fiber (cellulose-rich fractions) decreased from 60.31 to 39.65 kg with an increasing pretreatment temperature from 160°C to 200°C. The cellulose-rich fractions were then subjected to enzymatic hydrolysis. It was found that 26.38, 27.19, 28.63, and 25.14 kg glucose could be obtained when the hydrothermal pretreatment conditions were performed at 160°C for 60 min, 180°C for 30 min, 180°C for 60 min, and 200°C for 30 min, respectively. It should be noted that, after the hydrothermal pretreatment, the liquid and solid were separated by filtration and 1.24 to 7.65 kg xylo-oligosaccharides for 100 kg of initial fiber was observed in the hydrothermal liquid. Additionally, after subsequent alkali fractionation, 8.14 to 12.22 kg lignin for 100 kg of initial fiber was recovered, which could be converted into high-value materials. As we know, the viability of the two-step pretreatment process is strongly correlated to the glucose yield. However, the consumption of energy and the production of xylo-oligosaccharides and lignin should also be taken into account. Therefore, in this work, a combination of hydrothermal pretreatment at 180°C for 30 min and alkali treatment with 2% NaOH was considered as the optimum condition for glucose production and lignin recovery.

## Conclusions

Pretreatment is an important process for improving the digestibility of lignocellulosic biomass. During the hydrothermal pretreatment, it was found that hemicelluloses could be easily removed from the biomass as compared with cellulose and lignin. Moreover, alkali fractionation could significantly reduce the content of lignin in the biomass and remove part of the hemicelluloses. Both hydrothermal pretreatment and alkali fractionation could improve the enzymatic digestibility of eucalyptus fiber to some degree. More importantly, the combination of hydrothermal pretreatment and alkali fractionation resulted in a significant synergistic digestibility of eucalyptus fiber over that of the hydrothermal or alkali pretreatment alone. Under the optimal condition of hydrothermal pretreatment at 180°C for 30 min and sequent fractionation with aqueous alkali solution (2% NaOH, 90°C, 2.5 h), 27.19 kg fermentable glucose, 7.65 kg xylo-oligosaccharides, and 8.14 kg lignin for 100 kg of initial fiber could be recovered. The enzymatic hydrolysis rate of the treated fibers was enhanced 6.4 times by this combination of hydrothermal pretreatment and alkali fractionation compared to that of the raw material without treatment. Clearly, the combined treatment of hydrothermal pretreatment and alkali fractionation can be considered as a promising approach to achieve the efficient conversion of biomass to fermentable glucose for bioethanol production. Furthermore, the other two major components, xylo-oligosaccharides from hemicelluloses and the recovered lignin, can also be converted into high value-added products.

## Methods

### Raw materials

The thermo-mechanical fiber obtained at 120°C for 1 to 2 min from *Eucalyptus urophylla*, as raw material (RM), was kindly supplied by the State Key Laboratory of Pulp and Paper Engineering, South China University of Technology, China. The material was first dewaxed with methylbenzene/ethanol (2:1, v/v) in a Soxhlet apparatus for 3 h, and the sample was then dried in an oven for 12 h at 60°C for further use. The eucalyptus fiber consisted of 45.01% cellulose, 21.66% hemicelluloses, and 27.04% total lignin. All chemicals purchased were of analytical or reagent grade and used without further purification.

### Hydrothermal pretreatment and alkali fractionation

The hydrothermal pretreatment experiment was carried out in a laboratory-scale reactor (Parr Instrument Company, Moline, IL, USA) with a maximal volume of 1,000 ml. The feedstock 15.0 g was mixed with 450 ml of distilled water and then heated to 100 (60 min), 120 (60 min), 140 (60 min), 160 (60 min), 180 (15, 30, 45, and 60 min), 200 (30 min), 220 (30 min), and 240 (30 min)°C, respectively, at a heating rate of about 4°C/min. Once the desired operation was reached, the reactor was cooled to about 50°C by flowing water through an internal stainless steel loop. The liquid stream and solid residue were separated by filtration with a nylon cloth, and the residue was then washed thoroughly with hot water and dried in an oven at 60°C. The pretreated residues were labeled as R_100-60_, R_120-60_, AR_140-60_, R_160-60_, R_180-15_, R_180-30_, R_180-45_, R_180-60_, R_200-30_, R_220-30_, and R_240-30_, respectively, according to the pretreatment temperature and time. The liquid product was passed through 0.22-μm filters and stored at -50°C for further analysis. In alkali fractionation, the hydrothermal pretreated samples were further treated with 2% NaOH at 90°C for 2.5 h with a solid-to-liquid ratio of 1:30 (g/ml). The insoluble residues were collected by filtration, thoroughly washed and dried, and labeled as cellulose-rich fractions of AR_100-60_, AR_120-60_, AR_140-60_, AR_160-60_, AR_180-15_, AR_180-30_, AR_180-45_, AR_180-60_, AR_200-30_, AR_220-30_, and AR_240-60_, respectively. For comparison, un-pretreated material was also fractionated using the same alkaline solution and labeled as AM. Additionally, it should be noted that lignins were obtained during the alkali treatment, and the purification procedure of all lignin fractions was performed according to the method of a previous work [36].

### Analysis methods

The composition of the pretreated and un-pretreated samples was determined by the National Renewable Energy Laboratory (NREL) standard analytical procedure [37]. High-performance anion exchange chromatography (HPAEC) was used to quantify the sugars and uronic acids from the liquor obtained by H_2_SO_4_ hydrolysis of all the samples, using a Dionex ICS-3000 HPAEC system equipped with an AS50 autosampler and a CarboPac PA-20 column (4 × 250 mm, Dionex) [38].

The liquid obtained by hydrothermal pretreatment without dilution was filtered and stored to determine xylo-oligosaccharides and inhibitory products. A 3-ml liquid sample was subjected to quantitative post-hydrolysis with 4% H_2_SO_4_ at 121°C for 60 min. The increased concentration of monosaccharides caused by post-hydrolysis was regarded as the xylo-oligosaccharides concentration. The inhibitors were quantitatively measured on an HPLC system (Agilent 1200 series, Agilent Technologies, Santa Clara, CA, USA) equipped with a refractive index detector. The HPLC analysis was conducted using an HPX-87H ion exclusion column (with a length of 300 mm and an inner diameter of 7.8 mm, Bio-Rad, Laboratories, Hercules, CA, USA) operating at 50°C with 5 mM sulfuric acid at a flow rate of 0.6 ml/min.

The FTIR spectra of RM and pretreated residues were recorded on a Bruker spectrophotometer in the range of 400 to 4000 cm^-1^ with a resolution of 4 cm^-1^. A KBr disc containing 1% finely ground sample was used for measurement. XRD analyses of all samples were performed on a D8 Advance instrument (Bruker AXS) with Ni-filtered Cu Kα radiation (wavelength = 0.154 nm) from 5° to 60°. The CrI was measured from the XRD data and calculated with the following formula:

$$ \mathrm{CrI}=\frac{{\mathrm{I}}_{002}-{\mathrm{I}}_{\mathrm{am}}}{{\mathrm{I}}_{002}}\times 100 $$

Here I_002_ is the scattered intensity of cellulose I at about 2*θ* = 22.5°, and I_am_ is the peak for the amorphous portion assessed as the minimum intensity between the main and the secondary peaks at 2*θ* = 18.5°.

CP/MAS ^13^C NMR spectra of samples were obtained at 100.6 MHz using a Bruker AV-III 400 M spectrometer (Germany). Dried samples were packed in a 4-mm zirconia (ZrO_2_) rotor, and the measurements were performed using a CP pulse program with a 1-ms match time and a 2-s delay between transients. The spinning rate was 5 kHz. The SEM images were recorded with a Hitachi 3400 N scanning electron microscope operated at 10 kV and 81 mA. All samples were coated with gold prior to examination.

### Enzymatic hydrolysis

Enzymatic hydrolysis was conducted to comparably investigate the digestibilities of the raw material, hydrothermal pretreated fibers, and the cellulose-rich fractions obtained from the hydrothermal pretreatment and the subsequent alkali treatment. Enzymatic hydrolysis was carried out at 2% of substrate (w/v) in 10 ml of 50 mM sodium acetate buffer (pH 4.8) using a water bath shaking incubator at 150 rpm. The temperature was adjusted to 50°C for 72 h. The enzymes were cellulase and beta-glucosidase for all hydrolysis experiments, and the loadings were 17 FPU/g and 34 IU/g based on the dry weight of the substrate. The hydrolyzates were sampled periodically and analyzed by an HPAEC system (Dionex, ICS 3000, USA) on a CarboPac PA-100 analytical column. All enzymatic hydrolysis experiments were performed in duplicate, and the average values and corresponding deviations were given.

## Additional files

Additional file 1: Table S1. Chemical compositions of the raw material, hydrothermal pretreated fibers, and the cellulose-rich fractions. Additional file 2: Table S2. The concentrations of the inhibitors in the hydrothermal liquids. Additional file 3: Figure S1. FTIR spectra of the raw material, hydrothermal pretreated fibers, and the cellulose-rich fractions. Additional file 4: Figure S2. XRD patterns of the raw fiber, hydrothermal pretreated fibers, and the cellulose-rich fractions.

## Abbreviations

AIL: acid insoluble lignin
ASL: acid soluble lignin
CP/MAS ^13^C NMR: solid state cross-polarization/magic angle spinning ^13^C nuclear magnetic resonance
CrI: crystallinity index
FTIR: Fourier transform infrared spectroscopy
HPAEC: high-performance anion exchange chromatography
HPLC: high-performance liquid chromatography
NREL: National Renewable Energy Laboratory
RM: raw material
SEM: scanning electron microscopy
XRD: X-ray diffraction.
