# Supplementary material for: Improving the enzymatic hydrolysis of thermo-mechanical fiber from Eucalyptus urophylla by a combination of hydrothermal pretreatment and alkali fractionation
Source: Biotechnol Biofuels. 2014 Aug 20;7:116. doi: 10.1186/s13068-014-0116-8 (PMC4145232; doi:10.1186/s13068-014-0116-8)
Supplement: Additional file 1: Table S1. — Chemical compositions of the raw material, hydrothermal pretreated fibers, and the cellulose-rich fractions. [file 13068_2014_116_MOESM1_ESM.doc]

**Additional file 1: Table S1**

**Chemical compositions of the raw fiber, hydrothermal pretreated fibers and the cellulose-rich fractions obtained after the synergistic treatment.**

|  | Chemical composition (w/w, %) | | | |
| --- | --- | --- | --- | --- |
|  | Hemicelluloses | Cellulose | AIL^a^ | ASL^b^ |
| RM | 21.66±0.98 | 45.01±1.86 | 25.79±1.10 | 1.25±0.06 |
| R_100-60_ | 19.51±0.78 | 43.91±1.77 | 25.84±0.95 | 1.12±0.06 |
| R_120-60_ | 18.72±0.98 | 43.67±2.16 | 25.91±0.78 | 1.14±0.08 |
| R_140-60_ | 17.17±0.63 | 45.88±1.54 | 26.17±1.02 | 1.10±0.05 |
| R_160-60_ | 7.56±0.18 | 53.74±1.65 | 28.93±0.87 | 0.73±0.04 |
| R_180-15_ | 4.81±0.24 | 55.74±2.09 | 29.82±0.75 | 0.59±0.09 |
| R_180-30_ | 3.65±0.18 | 56.04±1.68 | 31.79±1.05 | 0.54±0.06 |
| R_180-45_ | 3.60±0.14 | 57.08±1.43 | 32.22±0.97 | 0.47±0.02 |
| R_180-60_ | 1.69±0.08 | 59.69±1.20 | 33.46±1.06 | 0.42±0.04 |
| R_200-30_ | 0.67±0.11 | 55.55±1.81 | 34.57±1.28 | ND |
| R_220-30_ | ND^c^ | 54.07±1.62 | 38.96±1.50 | ND |
| R_240-30_ | ND | 39.56±1.19 | 54.35±2.36 | ND |
| AM | 19.00±0.76 | 47.22±1.93 | 14.98±0.60 | 0.97±0.06 |
| AR_100-60_ | 13.28±0.53 | 50.31±1.51 | 15.06±0.75 | 0.96±0.06 |
| AR_120-60_ | 12.55±0.44 | 51.86±1.84 | 16.37±0.65 | 0.80±0.05 |
| AR_140-60_ | 9.41±0.38 | 58.73±1.76 | 17.05±0.80 | 0.71±0.04 |
| AR_160-60_ | 1.35±0.08 | 68.34±1.82 | 20.60±0.61 | 0.46±0.03 |
| AR_180-15_ | 0.22±0.01 | 69.68±2.09 | 20.73±0.83 | 0.43±0.03 |
| AR_180-30_ | 0.05±0.00 | 70.12±1.90 | 22.07±0.78 | 0.32±0.02 |
| AR_180-45_ | ND | 71.54±2.39 | 22.39±0.90 | 0.33±0.02 |
| AR_180-60_ | ND | 72.01±2.97 | 23.02±0.83 | 0.33±0.03 |
| AR_200-30_ | ND | 73.05±2.19 | 24.76±0.99 | 0.22±0.02 |
| AR_220-30_ | ND | 65.73±1.97 | 29.78±1.30 | 0.17±0.01 |
| AR_240-30_ | ND | 42.34±1.55 | 47.79±1.91 | 0.15±0.01 |

^a^ AIL was acid insoluble lignin.

^b^ ASL was acid soluble lignin.

^c^ Not detected.
