# Supplementary material for: Improving the enzymatic hydrolysis of thermo-mechanical fiber from Eucalyptus urophylla by a combination of hydrothermal pretreatment and alkali fractionation
Source: Biotechnol Biofuels. 2014 Aug 20;7:116. doi: 10.1186/s13068-014-0116-8 (PMC4145232; doi:10.1186/s13068-014-0116-8)
Supplement: Additional file 2: Table S2. — The concentrations of the inhibitors in the hydrothermal liquids. [file 13068_2014_116_MOESM2_ESM.doc]

**Additional file 2: Table S2. The concentrations** (g/L) of the inhibitors in the hydrothermal liquids.

| Temperature-Time (ºC-min) | Acetic acid (g/L) | HMF (g/L) | Furfural (g/L) |
| --- | --- | --- | --- |
| 100-60 | ND^a^ | ND | ND |
| 120-60 | 0.15 | ND | ND |
| 140-60 | 0.36 | ND | 0.01 |
| 160-60 | 0.58 | 0.02 | 0.03 |
| 180-15 | 0.32 | 0.04 | 0.06 |
| 180-30 | 0.62 | 0.05 | 0.14 |
| 180-45 | 0.78 | 0.06 | 0.16 |
| 180-60 | 1.23 | 0.07 | 0.23 |
| 200-30 | 1.89 | 0.25 | 0.73 |
| 220-30 | 2.36 | 0.70 | 0.97 |
| 240-30 | 3.28 | 1.27 | 1.45 |

^a^ Not detected.
