# Supplementary material for: Improving the enzymatic hydrolysis of thermo-mechanical fiber from Eucalyptus urophylla by a combination of hydrothermal pretreatment and alkali fractionation
Source: Biotechnol Biofuels. 2014 Aug 20;7:116. doi: 10.1186/s13068-014-0116-8 (PMC4145232; doi:10.1186/s13068-014-0116-8)

**Additional file 3: Figure S1.** FT-IR spectra of the raw material, hydrothermal pretreated fibers and the cellulose-rich fractions.


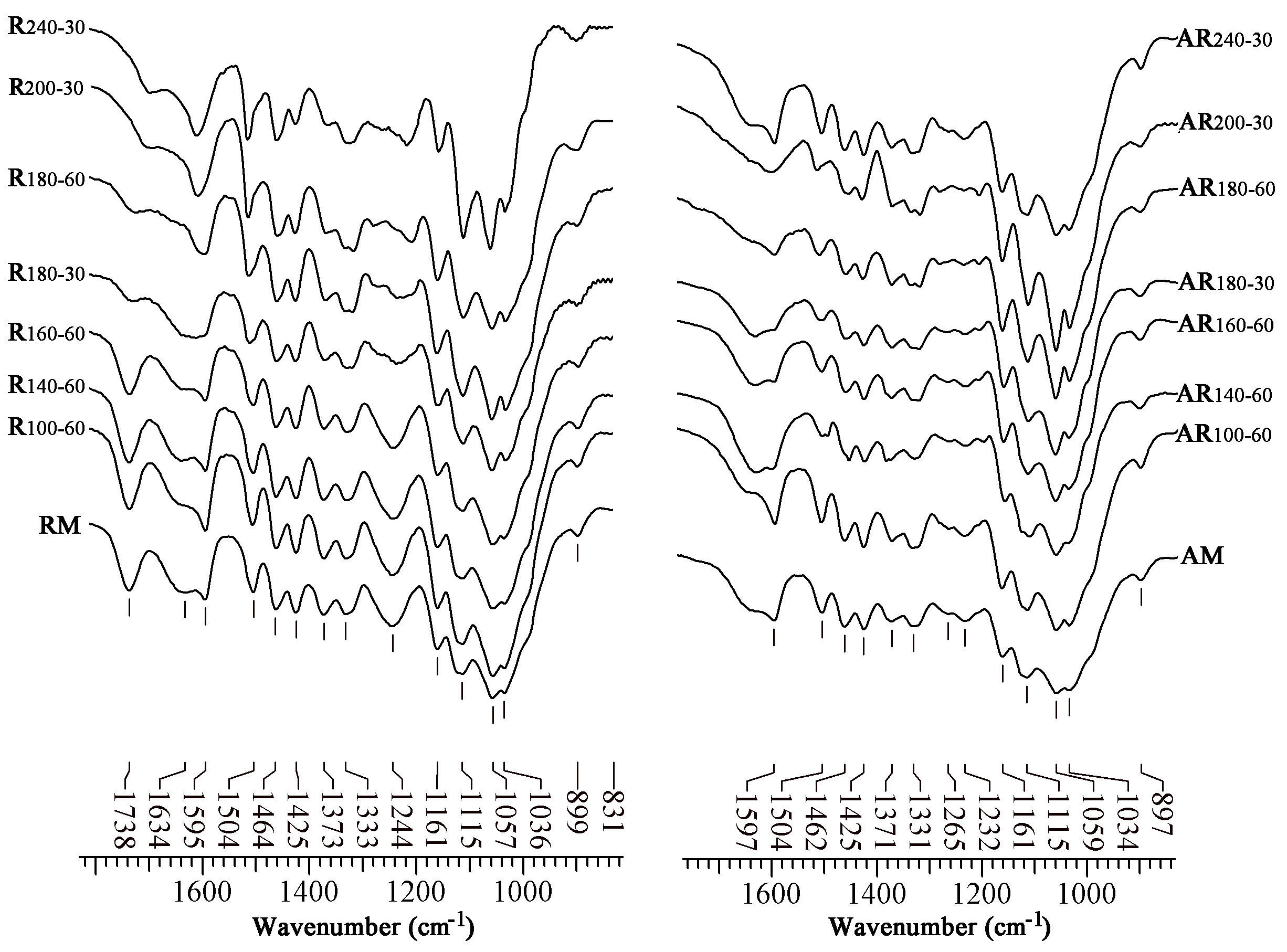

Supplement: Additional file 3: Figure S1. — FTIR spectra of the raw material, hydrothermal pretreated fibers, and the cellulose-rich fractions. [file 13068_2014_116_MOESM3_ESM.doc]
